# In Vivo Microendoscopy in the Near‐Infrared II Window

**DOI:** 10.1002/smll.202513882

**Published:** 2026-03-01

**Authors:** Zhisheng Wu, Danyang Xu, Zideng Dai, Xinyuan Wang, Wayne Jason Li, Sixin Xu, Xun Zhang, Yuanhua Liu, Puxian Xiong, Hanze Yu, Wentao Ye, Liangqiong Qu, Yongye Liang, Hongjie Dai, Feifei Wang

**Affiliations:** ^1^ Department of Electrical and Computer Engineering School of Biomedical Engineering The University of Hong Kong Hong Kong SAR China; ^2^ Materials Innovation Institute for Life Sciences and Energy (MILES) The University of Hong Kong‐SIRI Shenzhen China; ^3^ Department of Materials Science and Engineering Southern University of Science and Technology Shenzhen China; ^4^ Department of Mechanical Engineering The University of Hong Kong Hong Kong SAR China; ^5^ Department of Statistics and Actuarial Science The University of Hong Kong Hong Kong SAR China; ^6^ JC STEM Lab of Nanoscience and Nanomedicine Department of Chemistry and School of Biomedical Sciences The University of Hong Kong Hong Kong SAR China; ^7^ Department of Chemistry and Bio‐X Stanford University Stanford CA USA

**Keywords:** colitis, In vivo imaging, lymph node, microendoscopy, near‐infrared II imaging

## Abstract

Non‐invasive fluorescence endoscopy of deep biological tissues beyond the mucosa, or trans‐intestinal imaging in live mammals with high spatiotemporal resolution, remains challenging due to light scattering. Here, we developed a near‐infrared II (NIR‐II) microendoscopy with imaging wavelength extended to 1700 nm, enabling deep intestinal imaging beyond the mucosa and transrectal imaging at sub‐10‐µm resolution without any invasive surgery. It facilitated real‐time visualization of the relative motion of vascular networks across different layers of the mouse rectum. Featuring a specially designed large field of view, the NIR‐II microendoscopy enabled non‐invasive transrectal imaging of the entire lumbar lymph node for the first time, revealing abnormal lymphatic drainage in tumor‐bearing mice. In vivo longitudinal imaging of healthy mice and colitis‐bearing mice mapped distinct fluorescence patterns in rectal vasculature and lumbar lymph nodes, enabling observation of lesions in acute colitis at the initial stage, and opening new possibilities for studying the interaction between the intestinal microenvironment and lymphatic systems.

## Graphical abstract

NIR‐II microendoscopy with imaging wavelength extended to 1700 nm, enabling deep intestinal imaging beyond the mucosa and transrectal imaging at cellular resolution without any invasive surgery. It facilitates visualization of the relative motion of vascular networks, lymphatic drainage, and lesions in colitis models, opening new possibilities for studying the interaction between the intestinal microenvironment and lymphatic systems.

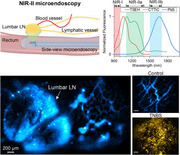

## 1 Introduction

As the body's largest digestive organ, the intestine is continuously exposed to foreign antigens, environmental agents from diet and microorganisms. It comprises the largest compartment of the immune system, containing extensive lymphoid tissues, protecting the body from pathogens while maintaining a stable internal environment [1, 2, 3]. The immune function of the intestinal region directly influences the progression of various diseases, and dysregulation of its microenvironment may lead to multiple disorders, including inflammatory bowel disease (IBD) [4], anxiety/depression symptoms [5], joint [6]/prostatic inflammation [7] or cancer [8].

In vivo imaging of mouse models has been widely applied to investigate intestinal diseases and associated immune responses [9]. Intravital imaging of murine intestines using confocal microscopy (488–561 nm excitation) [10] and two‐photon/multi‐photon microscopy (780–1040 nm excitation) [11, 12, 13, 14] has achieved penetration depths of < 100 and ∼100–300 µm, respectively. However, these methods rely on invasive intestinal externalization [10, 13] or surgically installed transparent windows [11, 15]. Microendoscopy offers an effective approach for non‐invasive or minimally invasive imaging of small animal intestines [9, 16, 17, 18, 19, 20, 21, 22, 23]. Visible (400–700 nm) wide‐field fluorescence microendoscopy allows for rapid superficial imaging of intestines [24], but its contrast and resolution are limited by strong light scattering and tissue autofluorescence [19, 25]. While structured illumination microendoscopy [19], near‐infrared confocal microendoscopy [26], and two‐photon/multi‐photon microendoscopy [20, 21] have achieved cellular resolution with high contrast, the imaging depth remains confined to the intestinal mucosa layer [16, 22, 23]. These microendoscopes face challenges in resolving the intestinal muscle layers or visualizing adjacent organs/tissues through the intact intestinal wall.

Recently, near‐infrared II (NIR‐II, 1000–3000 nm) fluorescence imaging has demonstrated deeper penetration, enhanced contrast, and improved resolution compared to conventional visible and near‐infrared I (NIR‐I, 700–900 nm) imaging owing to reduced light scattering and diminished tissue autofluorescence at longer wavelengths [27, 28, 29, 30, 31, 32, 33, 34, 35, 36, 37, 38, 39, 40, 41], enabling tissue penetration depths beyond 1 cm [42]. Numerous organic and inorganic NIR‐II fluorophores have been developed for vascular and hemodynamic imaging [34, 40, 43, 44, 45], calcium imaging [46], lymph node imaging [42, 47, 48, 49, 50, 51], nanorobot navigation [52], image‐guided surgery [53, 54, 55] and molecular imaging of tumors as well as immune responses [43, 47, 53, 56, 57]. NIR‐II light‐sheet microscopy (with excitation up to 1540 nm and emission up to 1700 nm) [57, 58] and NIR‐II confocal microscopy (using excitation up to 1650 nm and emission up to 2000 nm) [39, 59, 60] have enabled in vivo non‐invasive microscopy of blood vessels in the mouse head, tracking immune cells in the tumor microenvironment in response to immunotherapy, and molecular imaging of high endothelial venules, macrophages, and T cells within lymph nodes with cellular resolution. However, these NIR‐II imaging modalities are usually used to image animals from the external surface. It remains challenging to achieve cellular‐resolution imaging of internal lumens (e.g., the intestine) or tissues noninvasively.

Here, we present the first NIR‐II microendoscopy that utilizes small organic molecule dyes and quantum dots, extending the imaging wavelength to ∼1700 nm. NIR‐II microendoscopy facilitates non‐invasive visualization of intestinal and peri‐intestinal vasculatures and allows imaging of lumbar lymph nodes (LNs) through the intact intestinal wall with cellular resolution, benefiting from reduced light scattering and diminished tissue autofluorescence in the NIR‐II window. The field of view (FOV) of NIR‐II microendoscopy is optimized to ∼2.1 × 1.7 mm^2^ to capture the entire lumbar LN. NIR‐II microendoscopy allows for longitudinal in vivo imaging of healthy and 2,4,6‐trinitrobenzene sulfonic acid (TNBS)‐induced colitis‐bearing mice, mapping distinct fluorescence patterns in the rectum and descending colon, as well as in the lumbar LNs.

## 2 Results

### 2.1 NIR‐II Microendoscopy with a Large FOV

**FIGURE 1:**
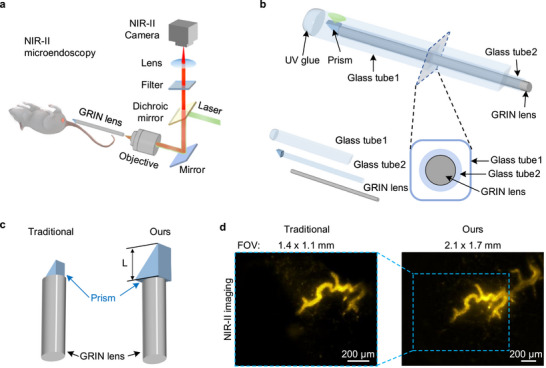
NIR‐II microendoscopy. (a) A schematic of the wide‐field NIR‐II microendoscope. (b) Configuration of the side‐view NIR‐II microendoscope. A prism was attached to the distal end of a glass tube (Glass tube 2), reflecting excitation illumination from the GRIN lens and fluorescence from the sample for side‐view imaging. For in vivo imaging, another rectangular glass tube (Glass tube 1), with a droplet of UV‐cured adhesive at the distal end to protect the mice, was first inserted into the rectum for endoscopic guidance. The GRIN microendoscope, encased in Glass tube 2, was then inserted into Glass tube 1, allowing for axial movement and rotation along the lumen. (c) Comparison of the configuration between traditional side‐view GRIN microendoscopy and our NIR‐II microendoscopy. The side lengths (L) of the prisms used in the traditional and our microendoscopes were 0.7 and 1.5 mm, respectively. (d) A comparison between traditional and our microendoscopy reveals a 1.5‐fold increase in FOV for ex vivo imaging of blood vessels in a fixed mouse rectum. The organic dye CTTIC was injected intravenously into a mouse. The mouse was euthanized ∼5 min post‐injection while the dye was still circulating in the blood vessels, and it was then fixed for ex vivo imaging. A 915‐nm laser was used for excitation, and fluorescence was collected after being filtered by a 1100‐nm long‐pass filter. The exposure time was 100 ms.

Our home‐built NIR‐II microendoscopic imaging system includes a NIR‐II wide‐field microscope and a gradient‐index (GRIN) lens‐based microendoscope (Figure 1a–c). The excitation laser beam was shaped via a lens set, reflected by a dichroic mirror, and focused near the proximal end of the GRIN microendoscope through a 10x objective (see Methods), generating wide‐field illumination at the sample. The NIR‐II fluorescence collected by the microendoscope and wide‐field microscope was recorded using an indium gallium arsenide (InGaAs) camera after being filtered by different filters (Figure 1a).

Traditional visible GRIN lens‐based microendoscopy enables in vivo fluorescence imaging of the mouse colon, small intestine, and esophagus, with a FOV smaller than 500 µm [9, 17, 18, 20], necessitating image stitching for large‐area imaging [9] and limiting real‐time observation of large, entire targets. For example, the size of the lumbar lymph nodes adjacent to the rectum measures ∼1.8 ± 0.3 mm (Figure S1). To image the entire lumbar LNs and facilitate large‐FOV in vivo imaging, we designed a doublet GRIN lens microendoscope with a 1‐mm diameter and a designed working distance of ∼5 mm (Methods) and attached an aluminum‐coated right‐angle prism to the GRIN objective for side‐view imaging (Figure 1b). The GRIN microendoscope was encased in a stainless protection sleeve with an outer diameter of ∼1.25 mm and a length of ∼45 mm. The commercially available GRIN microendoscope featured a prism with a side length of 0.7 mm (Figure 1c), resulting in a FOV of 1.4 × 1.1 mm^2^, smaller than the size of the lumbar LNs. When we replaced the small prism with a larger one with a side length of 1.5 mm, the FOV increased by 1.5 times to 2.1 × 1.7 mm^2^ without affecting the spatial resolution (Figure 1c,d) due to the increased optical path length and image collection area provided by the larger prism. To enhance flexibility, the larger prism was glued to the end of a glass tube (referred to as Glass tube 2 in Figure 1b, with an inner diameter of 1.35 mm and an outer diameter of 1.75 mm), which was then sleeved over the GRIN lens to create a side‐view microendoscope.

We quantitatively compared the FOV of a commercially available traditional side‐view microendoscope with our modified one by imaging vasculature in a fixed rectum (Figure 1d). We ensured that the distance between the side wall of the GRIN lens and the mouse intestine wall remained consistent (∼0.5 mm) during imaging. We intravenously injected an organic dye, CTTIC (excitation: 700–1100 nm; emission: 1000–1400 nm, Figure S2), into a mouse via the tail vein and euthanized the mouse ∼5 min post‐injection, when the dye was circulating in the vasculature. The mouse was fixed for ex vivo imaging. A 915‐nm laser was used to excite CTTIC, and the emission was filtered by a 1100‐nm filter before being recorded. The results demonstrated that our microendoscope improved the FOV by ∼50% compared to the traditional microendoscope, without compromising imaging performance (Figure 1d).

For in vivo imaging, mice were positioned in a supine posture on a motorized translation stage (Figure 1a). Another rectangular glass tube (referred to as Glass tube 1 in Figure 1b), with an inner side length of 2 mm and a droplet of UV‐cured adhesive at distal end to protect the mice, was inserted into the rectum. The GRIN microendoscope, encased in Glass tube 2, was then inserted into Glass tube 1, allowing for axial movement and rotation along the lumen while protecting both the prism and GRIN lens from tissue/intestinal secretion contamination, thus enabling clear imaging.

### 2.2 In Vivo Microendoscopy in NIR‐I, NIR‐IIa, and NIR‐IIb Regions

**FIGURE 2:**
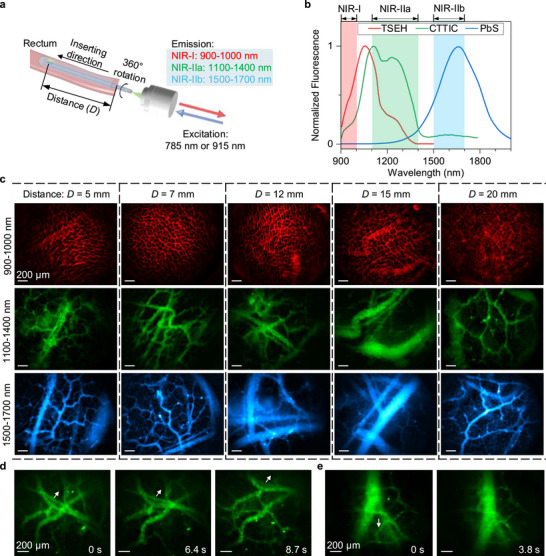
In vivo microendoscopy in NIR‐I, NIR‐IIa, and NIR‐IIb windows. (a) A simplified schematic of microendoscopy imaging of the mouse rectum. (b) Fluorescence emission spectra of TSEH, CTTIC, and PbS (Figure S2 shows excitation spectra). (c) Microendoscopy of the vasculatures at various distances (*D*) from the anus in the NIR‐I (900–1000 nm), NIR‐IIa (1100–1400 nm), and NIR‐IIb (1500–1700 nm) windows. The mice were injected intravenously with TSEH (*n* = 4), CTTIC (*n* = 7), and PbS (*n* = 3). *n* is the number of mice used for each group. A 785‐nm laser was used for TSEH excitation, and a 915‐nm laser was used for CTTIC and PbS excitation. (d,e) Time‐course recording of vasculature at different layers in the intestinal wall of mice injected with CTTIC (see Video S1). The white arrows indicate the movement direction of the vasculature over time. Relative motion between vasculatures at different layers was observed. A 915‐nm laser was used for excitation, and the emission was filtered by a 1100‐nm long‐pass filter before being recorded. The exposure time was 100 ms and the frame rate was ∼10 fps. Similar results for *n* > 3 independent experiments.

To compare microendoscopy imaging in different NIR‐I and NIR‐II spectral bands (Figure 2a), we employed three biocompatible NIR‐II probes (Figure S2), including TSEH (excitation/emission: 600–850 nm/900‐1300 nm) [61], CTTIC (excitation/emission: 700–1100/1000–1400 nm), and PEGylated PbS/CdS quantum dots (PbS, excitation/emission: ultraviolet‐1500/1400–1900 nm; Figure 2b) [39, 57, 59]. Following tail vein injection of these probes, the mice were immediately secured on a motorized translation stage for gradual microendoscope insertion. Non‐invasive NIR‐II microendoscopy of the rectum was performed while the probes were still circulating in the vasculature (Figure 2a).

We compared intestinal imaging of the mouse rectum at different distances (*D*, Figure 2a) from the anus in three fluorescence emission windows: 900–1000 nm (NIR‐I, TSEH emission, 785‐nm excitation), 1100–1400 nm (NIR‐IIa, CTTIC emission, 915‐nm excitation), and 1500–1700 nm (NIR‐IIb, PbS emission, 915‐nm excitation) (Figure 2b). The imaging results revealed that only the superficial capillary net with honeycomb‐shaped rings in the mucosa [62] was clearly resolved in the NIR‐I window, while deeper vasculature remained poorly visible, similar to previous observations using visible confocal microendoscopy [9, 63]. In the NIR‐IIa and NIR‐IIb windows, we observed vascular branches in the muscle layer deeper than the mucosa [62], and even outside the intestinal wall (Figure 2c). NIR‐II microendoscopy videos captured the relative movement of vasculatures across various layers within and outside the intestinal wall, driven by intestinal motility (Figure 2d,e; Video S1). The improvement in deep vascular imaging in the NIR‐II window is due to slower signal attenuation and reduced light scattering at longer wavelengths (scattering ∝ *λ* ^−^ *^k^*, where *λ* represents wavelength and *k* = 0.2–4.0 for biological tissues [30]). The signal attenuation was evaluated by imaging a 100 µm diameter capillary filled with TSEH (emission collection: 900–1000 nm, NIR‐I), CTTIC (emission collection: 1100–1400 nm, NIR‐IIa), or PbS (emission collection: 1500–1700 nm, NIR‐IIb), which was immersed at different depths in 1% intralipid solution. To avoid differences caused by excitation, all probes were excited with an 808‐nm laser. The results reveal that the NIR‐I signal decreases faster than the NIR‐IIa and NIR‐IIb signals as imaging depth < 8 mm (Figure S3). The lateral full width at half maximum (FWHM) values of the smallest intestinal vasculature in the NIR‐II window were 8.68 ± 0.26 µm (Figure S4), which is close to the theoretically estimated resolution (Note S1). The resolution of our NIR‐II microendoscope decreases slightly with increasing distance from the center of the field of view (Figure S5).

### 2.3 Non‐invasive NIR‐II Microendoscopy of Lumbar Lymph Nodes through the Intact Rectal Wall

Lumbar lymph nodes possess clinical and research significance, serving as common sites for metastasis due to their drainage function for abdominal, pelvic organs, and lower extremities [64, 65, 66]. Lumbar LNs are located between the rectum and the spine and extend along the paralumbar tissues [67] (Figure S1). It is difficult to observe the lumbar LNs using traditional intravital microscopy that requires invasive surgical implantation of imaging windows [11, 15], or with visible confocal/two‐photon/multi‐photon/NIR‐I microendoscopy, which is currently limited to imaging the mucosa layer [16, 20, 21, 22, 23].

**FIGURE 3:**
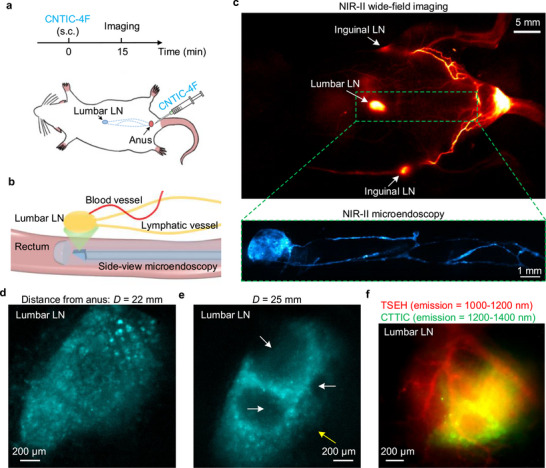
Transrectal NIR‐II microendoscopy of lumbar lymph nodes. (a) Top: subcutaneous (s.c.) injection of CNTIC‐4F and imaging schedule. Bottom: a schematic of subcutaneous injection of CNTIC‐4F (see Figure S2 for excitation and emission spectra) near the anus and lymphatic drainage to lumbar lymph nodes. (b) A schematic of in vivo microendoscopy performed on mice (*n* = 7). (c) Top: NIR‐II wide‐field imaging of a mouse 15 min after subcutaneous injection of CNTIC‐4F. Bottom: ex vivo NIR‐II microendoscopy of the lymphatic drainage network and connected lumbar lymph nodes. One hour after injection, mice were euthanized while CNTIC‐4F was still draining to lymph nodes, then they were fixed and preserved in glycerol for further ex vivo microendoscopy. We obtained the complete lymphatic drainage network, which drains CNTIC‐4F to the lumbar lymph node, by stitching together the imaging tiles. Ex vivo imaging was performed to eliminate the influence of intestinal motility. A 975‐nm laser was used for excitation, and fluorescence was filtered by a 1300‐nm long‐pass filter. (d,e) Lumbar LNs were recorded in the area 22 or 25 mm from the anus, 15 min after injecting CNTIC‐4F. A 975‐nm laser was used for excitation, and fluorescence was filtered by a 1200‐nm long‐pass filter instead of a 1300‐nm long‐pass filter to enhance the signal for real‐time imaging. The white and yellow arrows in (e) indicate the B cell follicles or T cell zones, respectively. (f) Two‐plex microendoscopy of the lumbar lymph node and surrounding blood vessels. TSEH was intravenously injected to label the vasculature, followed 5 min later by subcutaneous administration near the anus to label the lumbar LN. A 785‐nm laser and a 975‐nm laser were used to excite TSEH and CTTIC, respectively. The two‐plex NIR‐II microendoscopy was performed 15 min after the injection of CTTIC. The exposure time was 100 ms. Similar results for *n* > 3 independent experiments.

To perform NIR‐II microendoscopy of lumbar LNs, we subcutaneously injected CNTIC‐4F (excitation: 900–1300 nm; emission: 1200–1600 nm) [68] into the anal region (Figure 3a). At 15 min post‐injection of the probe, wide‐field NIR‐II imaging visualized bright signals in the inguinal LNs and lumbar LNs (Figure 3c), but the detailed structural information of the lumbar LNs cannot be resolved because of the limited resolution and the deep location of the lumbar LNs in the mouse body. Mice were secured in a supine position on a motorized stage for dorsally directed NIR‐II microendoscopy of CNTIC‐4F, exited at 975 nm, with emission filtered by a 1200‐nm long‐pass filter. At insertion depths of 4–8 mm from the anus, rectal crypts were observed with structures consistent with previous imaging using confocal laser endomicroscopy [22] (Figure S6). Advancing the imaging position to ∼22 mm, with a slight leftward rotation of the microendoscope, revealed one lumbar LN (Figure 3d). Further insertion to ∼25 mm, with a rightward rotation of microendoscope, exposed another lumbar LN (Figure 3e). We then conducted two‐plex imaging of blood vessels and lumbar LNs (Figure 3f). TSEH (excitation: 785 nm; emission: 1000–1200 nm) was intravenously injected to label vasculatures, followed 5 min later by the subcutaneous administration of CTTIC (excitation: 975 nm; emission: 1200–1400 nm) near the anus to label lumbar LNs. After 15 min, two‐plex NIR‐II microendoscopy revealed the relative positions between lumbar LN and blood vessels (Figure 3f). The B cell follicles or T cell zones can be clearly resolved [69] (Figure 3d–f), which are not observed in the wide‐field imaging (Figure 3c).

**FIGURE 4:**
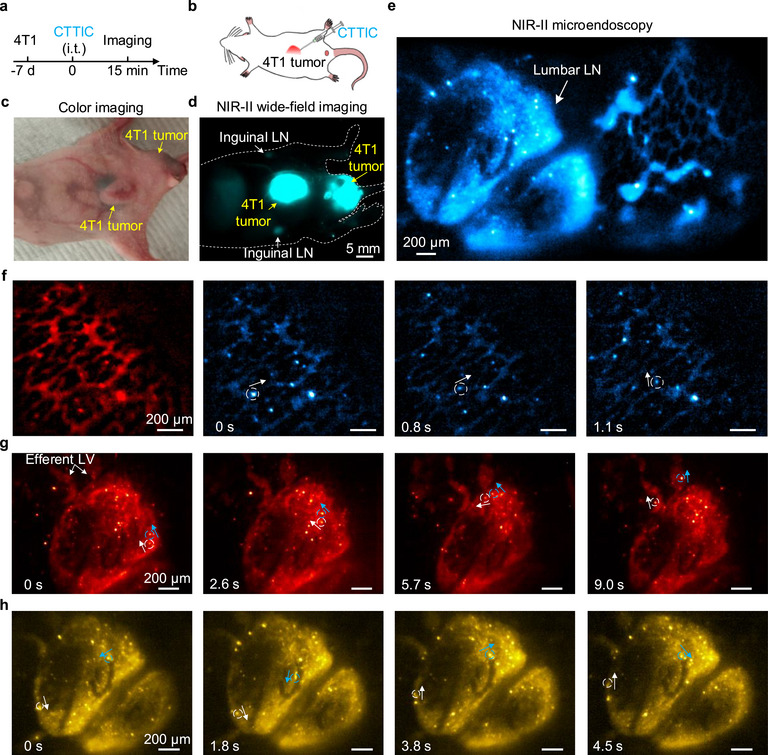
Non‐invasive NIR‐II microendoscopy of lumbar lymph nodes in tumor‐bearing mice. (a) Tumor inoculation, intratumoral injection, and imaging schedule. Two 4T1 breast tumors were subcutaneously inoculated in the abdominal region and near the anus. NIR‐II wide‐field imaging and microendoscopy of a mouse were performed 15 min after intratumoral injection of CTTIC (see Figure S2 for excitation and emission spectra). (b) A schematic of intratumoral injection of CTTIC. (c) Color photography and (d) NIR‐II wide‐field imaging of a mouse 7 days after tumor inoculation and 15 min after intratumoral injection of CTTIC. A 975‐nm laser was used for excitation, and fluorescence was filtered by a 1100‐nm long‐pass filter. (e) Non‐invasive in vivo NIR‐II microendoscopy of lumbar lymph nodes and surrounding lymphatic vessels. A 915‐nm laser was used for excitation, and fluorescence was filtered by a 1100‐nm long‐pass filter. The exposure time was 100 ms. (f) Time‐course imaging of the lymphatic vessel networks surrounding the lumbar LNs. The white arrows indicate the flow direction of CTTIC nanofluorophores moving in the lymphatic vessels. Time‐course recordings of (g) lymph flow through the lumbar LN (Video S2) and (h) abnormal lymph flows in the lumbar LN (Video S3). The white and blue arrows indicate the flow direction of representative two CTTIC nanofluorophores flowing to efferent lymphatic vessels (LV) or being transported with fluctuating patterns and reversal direction. (f–h) The frame rate was ∼10 fps.

NIR‐II microendoscopy enables in vivo imaging of lumbar LNs through the intact rectal wall, which has a thickness ranging from 300 to 600 µm in healthy mice (Figure S7). This capability facilitates the observation of the drainage from tumors to lumbar LNs non‐invasively. We subcutaneously inoculated two 4T1 breast tumors: one under the skin of the abdomen and the other near the anus (Figure 4c). Seven days after tumor inoculation, we intratumorally injected CTTIC nanofluorophores for lymphatic imaging (Figure 4a,b). At 15 min post‐injection, NIR‐II wide‐field imaging failed to visualize CTTIC drainage from 4T1 tumors to lumbar LNs via lymphatic vessels, as the tumor signal obscured the lumbar LNs signal (Figure 4d). Subsequent NIR‐II microendoscopy successfully captured real‐time CTTIC nanofluorophores (excitation: 915 nm; emission: 1200–1400 nm) transport within both lumbar LNs and the surrounding lymphatic vessels (Figure 4e–h; Videos S2 and S3). We observed lymphatic vessel networks surrounding the lumbar LNs (Figure 4e,f), with CTTIC nanofluorophores moving through the network at a speed of 150 ± 100 µm/s (Figure 4f). Time‐course imaging of the lumbar LNs revealed that lymph flows through the subcapsular sinus to the medullary sinuses at a speed of 120 ± 40 µm/s [70] and exits via efferent lymphatic vessels (Figure 4g; Video S2). Lymph flow in the lumbar LNs was observed to be irregular and intermittent, with fluctuating patterns and reversals of flow direction (Figure 4h; Video S3), which may be attributed to the lymphatic system dysfunction caused by tumor invasion [71]. The lateral FWHM values of the CTTIC nanofluorophores imaged using NIR‐II microendoscopy were 9.03 ± 1.01 µm (Figure S4), which is close to the theoretically estimated resolution at 1170 nm (Note S1), indicating that cellular resolution can be achieved.

### 2.4 In Vivo NIR‐II Microendoscopy of Mice With TNBS‐induced Colitis

Inflammatory bowel diseases (IBDs) can cause diarrhea and abdominal pain, and may lead to complications such as tissue fibrosis, stenosis, and colon cancer [72]. Novel imaging modalities aid in understanding the immunopathogenesis and early identification of IBDs, and in developing new therapeutic methods [73]. While in vivo imaging of colitis has also been achieved with visible confocal microendoscopy, its < 200 µm FOV and mucosal‐depth [74, 75, 76] limitation restricts the exploration of colitis‐lumbar LN interactions non‐invasively.

**FIGURE 5:**
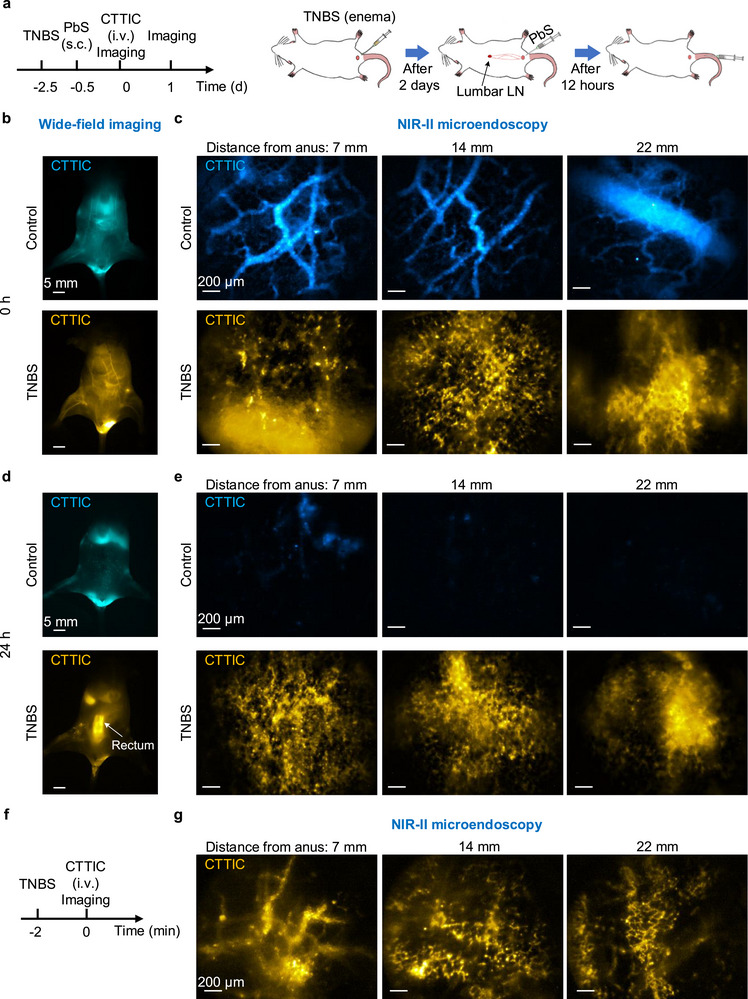
Longitudinal NIR‐II microendoscopy of the rectal vasculature in healthy and colitis‐bearing mice. (a) TNBS treatment, injection, and imaging schedule. Two days after intrarectal administration of TNBS, PbS was subcutaneously injected into the anal region of both healthy and TNBS‐treated mice to label lumbar LNs. Twelve hours later, CTTIC was injected intravenously into the mice through the tail vein. NIR‐II imaging was performed immediately and again one day post CTTIC injection. (b) NIR‐II wide‐field imaging was conducted immediately after the tail‐vein injection of CTTIC. The control group refers to healthy mice (*n* = 3), while the TNBS group refers to mice with TNBS‐induced acute colitis (*n* = 3). There was no apparent difference between healthy mice and TNBS‐treated mice in NIR‐II wide‐field imaging at this time point. A 975‐nm laser was used for CTTIC excitation, and its fluorescence was filtered by a 1100‐nm long‐pass filter and a 1400‐nm short‐pass filter. (c) In vivo NIR‐II microendoscopy of CTTIC in the rectum at different distances from the anus immediately after tail‐vein injection of CTTIC. The rectal blood vessels in healthy mice appeared normal, whereas strong, irregular fluorescence patterns due to CTTIC leakage were observed at multiple sites in the rectum of TNBS‐treated mice. (d) NIR‐II wide‐field imaging was conducted 24 h after tail vein injection of CTTIC, revealing strong fluorescence signals in the rectum of TNBS‐treated mice in contrast to healthy mice. (e) In vivo NIR‐II microendoscopy of the rectum of healthy and colitis‐bearing mice at different distances from the anus was performed 24 h post tail vein injection of CTTIC. The fluorescence signals in the rectum of healthy mice were negligible, whereas the signals in TNBS‐treated mice remained strong. Similar results for *n* = 3 independent experiments. (f) TNBS treatment, CTTIC injection, and microendoscopy schedule. (g) In vivo NIR‐II microendoscopy imaging at different distances from the anus was performed immediately after the intravenous injection of CTTIC following intrarectal administration of TNBS.

To demonstrate NIR‐II microendoscopy for IBDs, we performed longitudinal imaging of both healthy mice and mice with TNBS‐induced colitis (see Methods) [72]. On day 2 after intrarectal TNBS administration, PbS was subcutaneously injected into the anal region of both healthy and TNBS‐treated mice to label the lumbar LNs. Twelve hours later, CTTIC nanofluorophores were injected intravenously into these mice through the tail vein (Figure 5a).

Immediately after the injection of CTTIC, NIR‐II wide‐field imaging showed no apparent difference between healthy and colitis‐bearing mice (Figure 5b; Figure S8). However, NIR‐II microendoscopy revealed normal vasculature in healthy mice, whereas strong, irregular fluorescence patterns, induced by CTTIC leakage from vasculature [74], were observed at multiple sites in colitis‐bearing mice (Figure 5c). These irregular patterns indicated a disruption of the normal crypt architecture. It also demonstrated that CTTIC could visualize the lesions of IBDs.

At 24 h post‐injection of CTTIC, both NIR‐II wide‐field imaging and microendoscopy showed apparent differences between healthy and colitis‐bearing mice (Figure 5d,e). In contrast to the strong signal observed in colitis‐bearing mice, no obvious rectal signal was detected in healthy mice during wide‐field imaging (Figure 5d). NIR‐II microendoscopy revealed negligible signals in healthy mice, whereas colitis‐bearing mice exhibited strong, irregular fluorescence patterns that persisted without obvious reduction compared to the signal obtained immediately post‐injection (Figure 5e).

To validate the NIR‐II microendoscopy for the early detection of TNBS‐induced colitis, we intravenously injected CTTIC immediately after intrarectal administration of TNBS. Strong, irregular fluorescence patterns due to CTTIC leakage were observed at various sites in the rectum (Figure 5f,g; Figure S9), which were different from those of healthy mice, indicating that TNBS induced rapid capillary damage post administration (Figure 5c). NIR‐II microendoscopy enables the earliest detection of TNBS‐induced acute colitis that can not achieved by traditional methods [72, 77].

**FIGURE 6:**
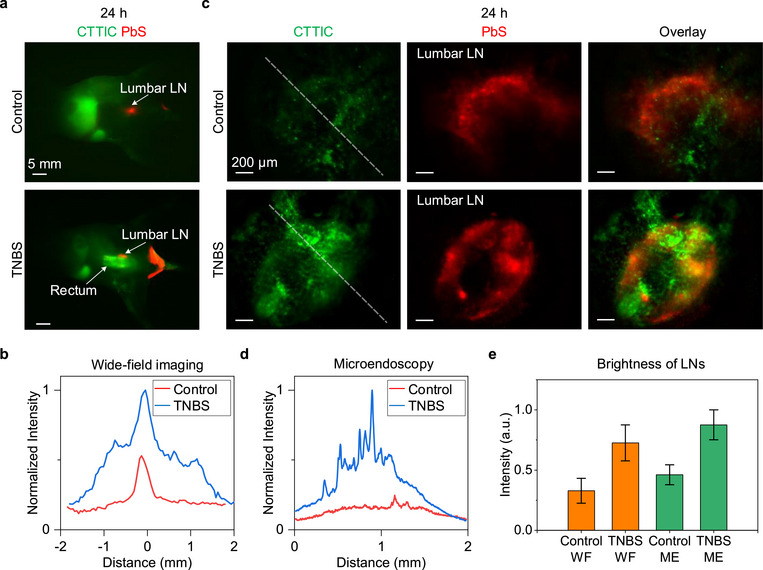
Transrectal NIR‐II microendoscopy of the lumbar lymph nodes in healthy and colitis‐bearing mice. (a) NIR‐II wide‐field two‐plex imaging (red: PbS, excitation: 808 nm, emission: 1500–1700 nm; green: CTTIC, excitation: 975 nm, emission: 1100–1400 nm) of healthy (*n* = 3) and colitis‐bearing mice (*n* = 3) 24 h after tail‐vein injection of CTTIC. The treatment, injection, and imaging schedule are shown in Figure 5a. (b) Normalized intensity profiles across the lumbar LNs in the dissected mice are shown in (a). Mice were sacrificed after all in vivo imaging and dissected to expose the lumbar LNs (Figure S10). Wide‐field imaging of the exposed lumbar LNs was performed to compare fluorescence intensity, preventing the influence of the tissues above the lumbar LNs on fluorescence signals. The data were normalized to the maximum value of the intensity profile across the lumbar lymph nodes in the TNBS mice. (c) In vivo two‐plex transrectal NIR‐II microendoscopy of the lumbar LNs in healthy and colitis‐bearing mice was conducted 24 h after tail vein injection of CTTIC (red: PbS, excitation: 808 nm, emission: 1500–1700 nm; green: CTTIC, excitation: 915 nm, emission: 1100–1400 nm). (d) Normalized intensity profiles along the white‐dashed lines in (c). The data were normalized to the maximum intensity value across the lumbar lymph nodes in TNBS‐treated mice. (e) The fluorescence intensity of the lumbar lymph nodes in healthy and colitis‐bearing mice was recorded using the ex vivo NIR‐II wide‐field (WF) imaging system and in vivo microendoscope (ME). The intensity data is shown as the mean ± S.D. Bar charts represent the mean values, while error bars represent s.d. for n  =  3.

We further conducted imaging of CTTIC in the lumbar LNs one day post the CTTIC injection (Figure 6). Non‐invasive two‐plex NIR‐II wide‐field imaging (Figure 6a) and microendoscopy (Figure 6c) using CTTIC (excitation: 915 nm; emission: 1100–1400 nm) and PbS (excitation: 808 nm; emission: 1500–1700 nm; injected subcutaneously near the mouse anus to label lumbar LNs) facilitated the localization of lumbar lymph nodes in these imaging modalities. However, from the wide‐field imaging, we did not observe CTTIC signals in the lumbar LNs in vivo in either control or TNBS‐treated mice, while these signals were resolved by NIR‐II microendoscopy, revealing that NIR‐II microendoscopy provides a more effective way to achieve high‐resolution imaging of weak signals in internal lumens or tissues. To compare traditional NIR‐II wide‐field imaging with microendoscopy, we measured the signals of lumbar LNs from dissected mice after euthanasia (Figure 6b and Figure S10). Both ex vivo NIR‐II wide‐field imaging and non‐invasive in vivo NIR‐II microendoscopy revealed that the CTTIC signals in colitis‐bearing mice were about twice as high as those in healthy mice (Figure 6b–e), which can be attributed to the leakage of CTTIC at lesion sites draining into the lumbar lymph nodes.

## 3 Conclusion

This work developed NIR‐II microendoscopy, extending the imaging wavelength of endoscopy to ∼1700 nm for non‐invasive in vivo imaging of internal lumens (e.g., the intestine) or tissues with single blood vessel or cellular resolution. NIR‐II microendoscopy effectively suppressed light scattering and tissue autofluorescence, enabling clear visualization of blood vessels in the intestinal muscle and peri‐intestinal layers beyond the mucosa, which is challenging for traditional microendoscopy [9, 16, 19, 20, 21, 22, 23, 24, 25], confocal microendoscopy [74, 75, 76], and intravital imaging modalities [10, 11, 12, 13, 14, 15]. For the first time, we achieved non‐invasive, high‐resolution imaging of lumbar LNs through the intact intestinal wall using NIR‐II microendoscopy with an optimized FOV larger than previous microendoscopes. Relative movement of vasculature across different intestinal layers, driven by intestinal motility, and irregular lymph flow in the lumbar LN were observed in real time. NIR‐II microendoscopy enabled the longitudinal investigation of intestinal lesions and their drainage to the lumbar LNs in a TNBS‐induced IBD model with single blood vessel resolution. These observations are difficult to achieve by traditional NIR‐II imaging modalities, such as conventional wide‐field imaging [42, 47, 48, 49, 50, 51], confocal microscopy [39, 59, 60], light‐sheet microscopy [57, 58], and other existing endoscopes (Table S1). Compared with recently reported NIR‐II endoscopy operating at ≥ 900 nm [78], our NIR‐II microendoscope enables better resolution, side‐view imaging capability, whole‐LN FOV engineering, a longer working wavelength, and the capability for imaging through the intact intestinal wall (Table S1).

We have currently implemented NIR‐II microendoscopy in wide‐field mode, which offers ease of operation and a fast frame rate but lacks optical sectioning capability. It could be further advanced by developing NIR‐II confocal microendoscopy to impart optical sectioning capability for three‐dimensional imaging. Real‐time imaging at higher frame rates could be achieved by using cameras with greater sensitivity or fluorescence probes with higher quantum yields. The trade‐off between FOV and resolution should be carefully considered, as larger FOV typically comes at the expense of a reduced resolution. The introduction of structured illumination [58] and super‐resolution artificial intelligence (AI) networks provides options to enhance resolution and reduce background noise. Extending the imaging wavelength beyond 1700 nm could further reduce light scattering, improve imaging contrast, and increase penetration depth.

## 4 Methods

### 4.1 NIR‐II Microendoscope Setup

We fabricated a side‐viewing microendoscope using a 1‐mm‐diameter GRIN lens (GoFoton America) and an aluminum‐coated right‐angle microprism with a side length of 1.5 mm (Figure 1c). The lens assembly adopted a dual‐lens configuration, comprising a proximal coupling lens (CLH, pitch = 0.278) and a relay lens (SRL, pitch = 1). The designed working distance of the dual‐lens microendoscope was 5 mm, enabling clear imaging at actual working distances from ∼1 mm to more than 5 mm by adjusting its proximal end relative to the objective. The assembled dual‐lens microendoscope was inserted into a stainless‐steel sheath and secured with adhesive, leaving a 0.5‐mm lens protrusion at the CLH end. The microprism was glued to the end of a glass tube (Glass tube 2, outer diameter: 1.75 mm, inner diameter: 1.35 mm), which was then sleeved over the GRIN lens to enable side‐view imaging. A rectangular glass tube (Glass tube 1, inner side length: 2 mm) was sealed at one end with UV‐curable adhesive (NOA63, Thorlabs) and inserted into the mouse anus as a protective transparent channel for the microendoscope (Figure 1b).

The laser was delivered to the beam‐shaping module via an optical fiber (M28L02, Thorlabs), reflected by a dichroic mirror, focused with a 10× objective (LMPlan NIR 10×, Olympus), and coupled into the GRIN microendoscopy to generate uniform wide‐field illumination. Fluorescence was collected by the GRIN microendoscope and the wide‐field microscope, filtered by different filters, and then recorded by an InGaAs camera (C‐RED 2, Oxford Instruments). A 200‐mm tube lens (TTL200‐S8; Thorlabs) was used in the wide‐field microscope. The 10× objective was mounted on a translation stage, and precise focusing of the GRIN microendoscope image plane was achieved by adjusting the translation stage to control the distance between its proximal end and the objective (Figure 1a). The full FOV of the GRIN lens is circular, while the images captured by the camera are rectangular (2.1 mm × 1.7 mm), as we collected only the signal from the central portion of the GRIN lens FOV to fully utilize the camera's pixels for sample imaging. The light path was not changed when we performed two plex imaging, except that the excitation laser and emission filters were quickly switched. The excitation power was measured by a power meter (TS15+TP100, CNI Laser). For in vivo microendoscopy, the actual excitation illuminated on the mouse were approximately 50, 100, 120, and 110 mW/cm^2^ for 785, 808, 915, and 975 nm lasers, respectfully.

To compare the field‐of‐view differences induced by prism size, we performed ex vivo imaging of blood vessels in a fixed mouse rectum, as shown in Figure 1c,d. The organic dye CTTIC was injected intravenously into a mouse. The mouse was euthanized ∼5 min post‐injection while the dye was still circulating in the blood vessels, and it was then fixed for ex vivo imaging. A 915‐nm laser was used for excitation and fluorescence was collected after being filtered by a 1100‐nm long‐pass filter. During imaging, we ensured that the distance between the GRIN lens sidewall and the mouse intestinal wall was the same (∼0.5 mm). The two types of microendoscopes were then used sequentially to capture images of blood vessels at the same intestinal location in order to compare the field of view.

### 4.2 NIR‐II Fluorescent Probes

This study employed organic small‐molecule dyes, including TSEH [61], CNTIC‐4F [68], CTTIC, and PbS quantum dots [39, 59] (see absorption and emission spectra in Figure S2). TSEH exhibits an absorption peak near 760 nm, with emission from 900 to 1300 nm and an emission peak at 1050 nm. Because a portion of its emission spectrum falls within the 900–1000 nm band (Figure S2a), fluorescence imaging performance in the NIR‐I region can be evaluated using appropriate optical filters. CNTIC‐4F has an absorption peak at 1012 nm and a broad emission spectrum peaking at 1300 nm. Its relatively broad emission spectrum ranges from 1200 to 1600 nm (Figure S2b), providing higher clarity and improved imaging performance for lymphatic system imaging in the NIR‐IIb window. CTTIC displays a broad absorption spectrum centered near 1000 nm and emits across 1100–1400 nm, corresponding to the NIR‐IIa window, and can be used to evaluate fluorescence imaging in this region. Dynamic light scattering (DLS) measurements revealed that the average hydrodynamic size of CTTIC nanofluorophores was 60 nm. PbS has a wide absorption range (ultraviolet–1500 nm) and emits from 1500 to 1900 nm, suitable for assessing fluorescence imaging in the NIR‐IIb region.

### 4.3 Mouse Handling and Tumor Inoculation

All animal experiments were approved by the Committee on the Use of Live Animals in Teaching and Research (CULATR) at The University of Hong Kong (approval number: 23‐016). Female C57BL/6 mice, BALB/c mice, and BALB/c nude mice were obtained from the Centre for Comparative Medicine Research (CCMR) of the University of Hong Kong. Bedding, nesting materials, food, and water were provided, with relative humidity maintained at 55%–65% and temperature at approximately 25°C. Prior to NIR‐II in vivo imaging, mice were anesthetized using a rodent anesthesia machine with a flow rate of 3 L/min of oxygen mixed with 2% isoflurane. During imaging, anesthesia was maintained via a nose cone delivering a mixture of 1.5 L/min oxygen and 0.3% isoflurane. All mice used in the experiments were randomly selected from their cages. For in vivo imaging, the mouse hair was removed using depilatory cream.

### 4.4 Preparation of Mice with TNBS‐induced Colitis

We mixed 50 µL of 5% TNBS solution with 50 µL of absolute ethanol, then loaded the prepared mixture into a 1‐mL syringe fitted with a gavage needle. After anesthetizing the mice, we inserted the gavage needle ∼3 cm into the rectum via the anus and slowly injected the TNBS solution. The catheter was then gently withdrawn from the rectum, and the mouse was maintained in a head‐down vertical position for 60 s.

### 4.5 Preparation for In Vivo Microendoscopy

Before in vivo microendoscopy, the colon and rectum were flushed and lubricated with 0.2 mL of PBS buffer, which was injected via a small pipette tip to minimize tissue damage. Square Glass tube 1 was disinfected with 75% ethanol, and a small drop of PBS was applied to its tip to facilitate insertion into the mouse intestine. The microendoscope was also sterilized with 75% ethanol before being advanced into the square glass tube. The mice were placed on a motorized translation stage (MTS50‐Z8; Thorlabs), and the motorized translation stage was connected to a manual XYZ translation stage. The XYZ translation stage was adjusted so that the square Glass tube 1 inside the anus was fully aligned and parallel to the microendoscope. The Glass tube 2 attached with prism can be rotated within the Glass tube 1 to observe different directions, and the microendoscope can gradually enter the mouse rectum using the motorized translation stage (Figure 1a).

### 4.6 In Vivo NIR‐II Microendoscopy of Lumbar LNs

For in vivo imaging of the lumbar LNs as shown in Figure 3d–f, 50 µL of CNTIC‐4F with an OD of 20 at 808 nm was subcutaneously injected near the mouse anus. CNTIC‐4F was preferred for in vivo imaging as it does not contain toxic elements such as Pb in PbS. After 15 min, the microendoscope was inserted into the rectum for more than 20 mm, and the prism was oriented toward the spinal direction for observation. The lumbar LNs were initially located within a ± 30° range relative to the spinal direction, and the prism could be rotated to locate them.

For non‐invasive NIR‐II microendoscopy of tumor draining lumbar LNs as shown in Figure 4, 4T1 tumors near the abdomen and anus were induced in 4‐ to 6‐week‐old BALB/c nude mice via subcutaneous injection of 1 × 10^7^ cells suspended in 100 µL PBS. Seven days after inoculation, 100 µL of CTTIC with an OD of 10 at 808 nm was injected intratumorally. NIR‐II microendoscopy was performed 15 min post‐injection of CTTIC. A 915‐nm laser was used for excitation and a 1100‐nm long‐pass filter was used to filter the fluorescence. The exposure time was 100 ms and the frame rate was ∼10 fps.

For longitudinal in vivo NIR‐II microendoscopy of healthy and TNBS‐induced colitis‐bearing mice, as shown in Figures 5 and 6. Two days after administration of TNBS, 50‐µL PbS with an OD of 8 at 808 nm was injected subcutaneously near the mouse anus. Twelve hours later, 200‐µL CTTIC with an OD of 10 at 808 nm was injected intravenously via the tail vein. NIR‐II microendoscopy was performed immediately and again 24 h after CTTIC injection. An 808‐nm laser and a 915‐nm laser were used for PbS and CTTIC excitation, respectively. The emissions from PbS and CTTIC were collected in the windows of 1500–1700 and 1100–1400 nm, respectively.

### 4.7 In Vivo NIR‐II Wide‐field Imaging

In vivo NIR‐II wide‐field fluorescence images were recorded by a two‐dimensional InGaAs camera. For the CNITC‐4F channel in Figure 3c, excitation light was provided by a 975‐nm laser, and fluorescence was collected after being filtered by a 1300‐nm long‐pass filter. The exposure time was 20 ms. For the imaging of CTTIC shown in Figure 4d, excitation light was provided by a 975‐nm laser, and fluorescence was collected after being filtered by a 1100‐nm long‐pass filter. The exposure time was 20 ms. For two‐plex imaging shown in Figure 6a, CTTIC and PbS were excited by a 975‐nm laser and an 808‐nm laser, respectively. The fluorescence of CTTIC and PbS was collected at 1100–1400 nm (filtered with a 1100‐nm long‐pass filter and a 1400‐nm short‐pass filter) and 1500–1700 nm (filtered with a 1500‐nm long‐pass filter), respectively.

### 4.8 Statistical and Data Analysis

The raw data of microendoscopy were processed in ImageJ (1.54p). Motion artifacts were corrected using the “Linear Stack Alignment with SIFT” plugin in Fiji (1.54p). Two‐plex fluorescence image merging was also performed in ImageJ. Data analysis was performed using MATLAB (2022) or OriginLab (2021). The mean values and standard deviations presented in Figure 6 were calculated using OriginLab (2021).

## Author Contributions

F.W., H.D. and Z.W. conceived and designed the experiments. F.W. and Z.W. designed the optical system. Z.W. set up the optical system. Z.W., D.X. and Z.D. performed the experiments. Z.D. prepared the PbS/CdS quantum dots. X.W. and Y.L. provided the CTTIC and TSEH. X.Z. and Y.L. provided the CNTIC‐4F. Z.W., D.X., Z.D., X.W., W.J.L., S.X., X.Z., Y.L., H.Y., W.Y., L.Q., Y.L., H.D. and F.W. analyzed the data. Z.W., D.X., Z.D. and F.W. wrote the manuscript. All authors contributed to the general discussion and revision of the manuscript.

## Conflicts of Interest

The authors declare no conflicts of interest.

## Supporting information




**Supporting File 1**: smll72978‐sup‐0001‐SuppMat.pdf.

**Supporting File 2**: smll72978‐sup‐0002‐VideoS1.avi.

**Supporting File 3**: smll72978‐sup‐0003‐VideoS2.avi.

**Supporting File 4**: smll72978‐sup‐0004‐VideoS3.avi.

## Data Availability

The data that support the findings of this study are available from the corresponding author upon reasonable request.
