# Supplementary material for: In Vivo Microendoscopy in the Near‐Infrared II Window
Source: Small. 2026 Mar 1;22(24):e13882. doi: 10.1002/smll.202513882 (PMC13114502; doi:10.1002/smll.202513882)
Supplement: Supplementary file 1 — Supporting File 1: smll72978‐sup‐0001‐SuppMat.pdf. [file SMLL-22-e13882-s001.pdf]

Supplementary Materials for

In Vivo Microendoscopy in the Near-Infrared II Window

Zhisheng Wu[1,2,†], Danyang Xu[1,†], Zideng Dai[1,†], Xinyuan Wang[3], Wayne Jason Li[4], Sixin Xu[1], Xun Zhang[3], Yuanhua Liu[1], Puxian Xiong[1], Hanze Yu[1], Wentao Ye[1], Liangqiong Qu[5], Yongye Liang[3,]*, Hongjie Dai[2,4,6,7,]* and Feifei Wang[1,2,]*

*Correspondence to: liangyy@sustech.edu.cn, hjdai@hku.hk, feifwang@hku.hk

SUPPLEMENTARY VIDEO CAPTIONS

Video S1 | Time-course recording of vasculature at different layers in the intestinal wall of mice injected with CTTIC intravenously. A 915-nm laser was used for excitation, and fluorescence was filtered by an 1100-nm long-pass filter. We observed relative motion between vasculature at different layers. The exposure time was 100 ms and the frame rate was ~ 10 fps.

Video S2 | Time-course recording of lymph flow through the draining lumbar lymph node. CTTIC nanofluorophores were injected intratumorally seven days after inoculation of 4T1 tumors. NIR-II microendoscopy was performed 15 minutes post-injection of CTTIC. A 915-nm laser was used for excitation, and fluorescence was filtered by an 1100-nm long-pass filter. The motion of CTTIC nanofluorophores flowing through the subcapsular sinus to the medullary sinuses at a speed of 120 ± 40 μm/s, and exiting via efferent lymphatic vessels, was observed. The exposure time was 100 ms and the frame rate was ~ 10 fps.

Video S3 | Time-course recording of abnormal lymph flows in the lumbar lymph node. NIR-II microendoscopy was performed 15 minutes post intratumoral injection of CTTIC and seven days after inoculation of 4T1 tumors. A 915-nm laser was used for excitation, and fluorescence was filtered by an 1100-nm long-pass filter. The motion of CTTIC nanofluorophores exhibiting fluctuating patterns and reversals of direction was observed. The exposure time was 100 ms and the frame rate was ~ 10 fps.

Note S1. Theoretical resolution of NIR-II microendoscopy

The diffraction-limited resolution (R) of the NIR-II microendoscope can be estimated using Rayleigh criterion, $R = 0.61\lambda/\text{NA}$, where λ is the working wavelength and NA is the numerical aperture of the GRIN lens of the NIR-II microendoscope. For the NIR-II microendoscope used in this study, the NA is ~ 0.085, enabling a theoretical resolution of ~ 7.18 μm, ~ 8.40 μm and ~ 12.2 μm at wavelengths of 1000 nm, 1170 nm and 1700 nm, respectively. These estimated values are close to the FWHM values of the smallest intestinal vasculature (FWHM = 8.68 ± 0.26 μm) and of the CTTIC nanofluorophores in the lumbar LN (FWHM = 9.03 ± 1.01 μm).

SUPPLEMENTARY FIGURES

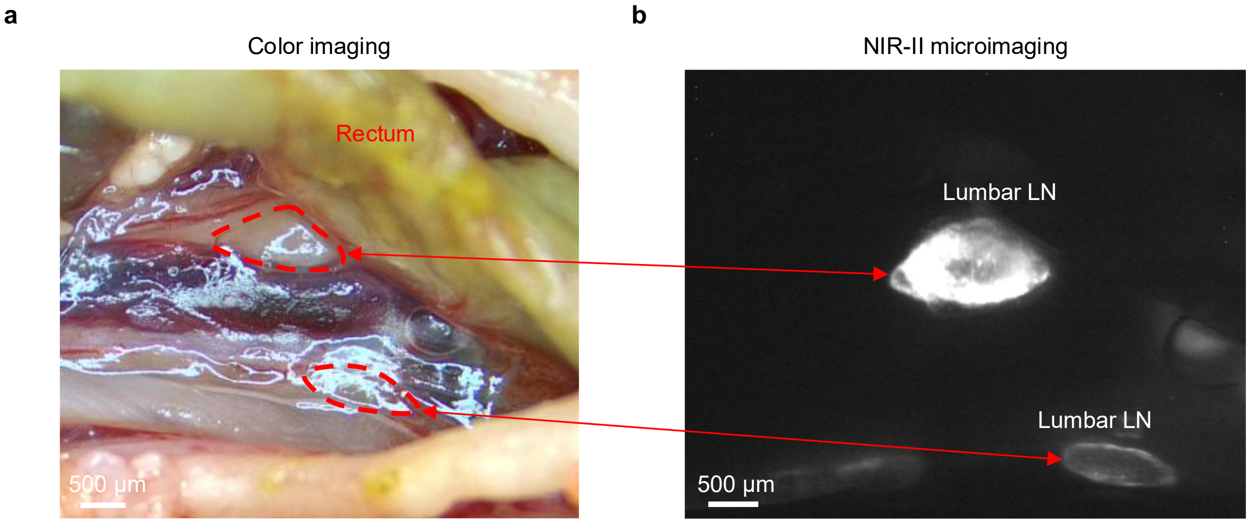

Figure S1. Ex vivo imaging of lumbar LNs. (**a**) Color imaging and (**b**) NIR-II microscopy of lumbar LNs. PbS was subcutaneously injected into the anal region of mice (n = 5), which were then euthanized 24 hours post-injection. The imaging results revealed that the lumbar LNs are located between the rectum and the spine, with a size of ~ 1.8 ± 0.3 mm. An 808-nm laser was used for excitation of PbS, and fluorescence was filtered by a 1500-nm long-pass filter. The lumbar LNs show strong NIR-II fluorescence signals. Scale bars, 500 µm. Similar results for $n > 3$ independent experiments.

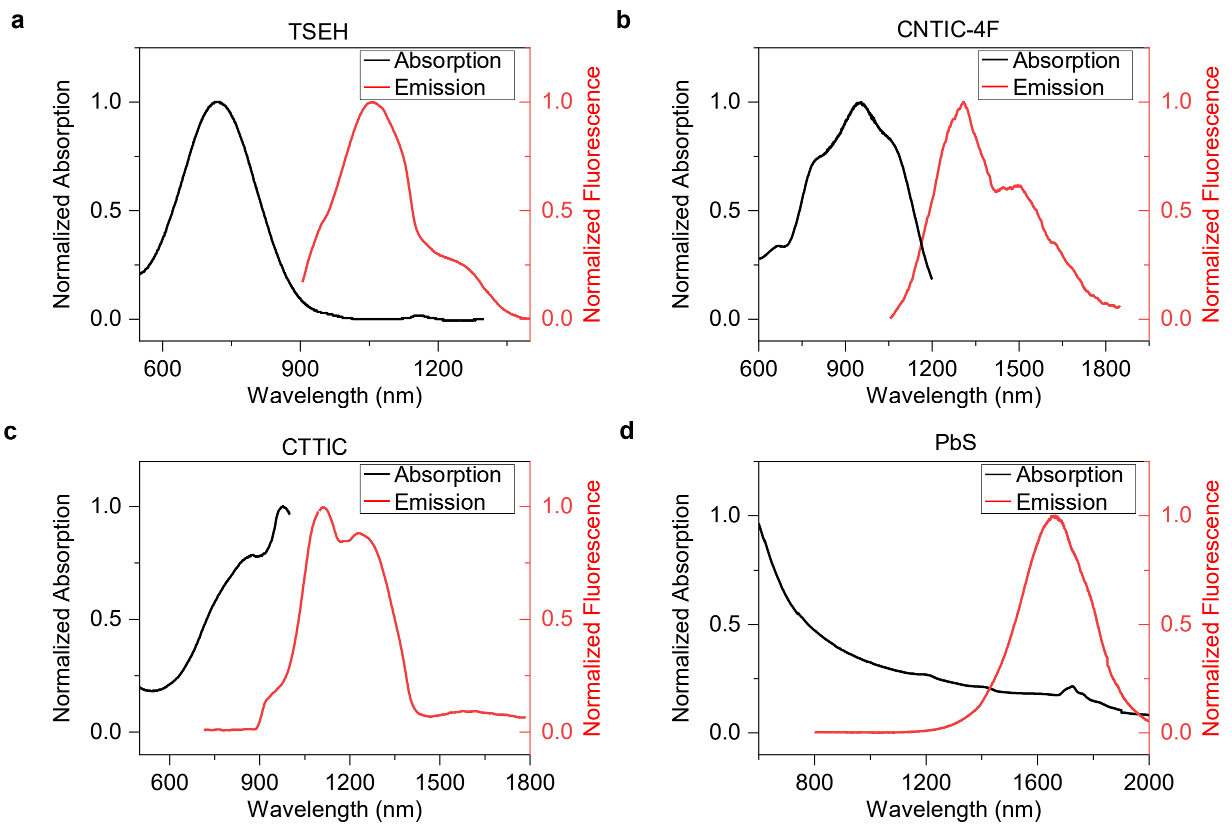

Figure S2. Absorption and emission spectra of (**a**) TSEH, (**b**) CNTIC-4F, (**c**) CTTIC, and (**d**) PbS. Taking advantage of TSEH, CTTIC, and PbS, we were able to image the rectal vasculature in three fluorescence emission windows: 900-1000 nm (NIR-I, TSEH emission), 1100-1400 nm (NIR-IIa, CTTIC emission), and 1500-1700 nm (NIR-IIb, PbS emission) respectively. CNTIC-4F was subcutaneously injected into the anal region of mice for lumbar LN imaging. By selecting suitable lasers and filters, two-plex microendoscopy of vasculature and lumbar LNs could be achieved.

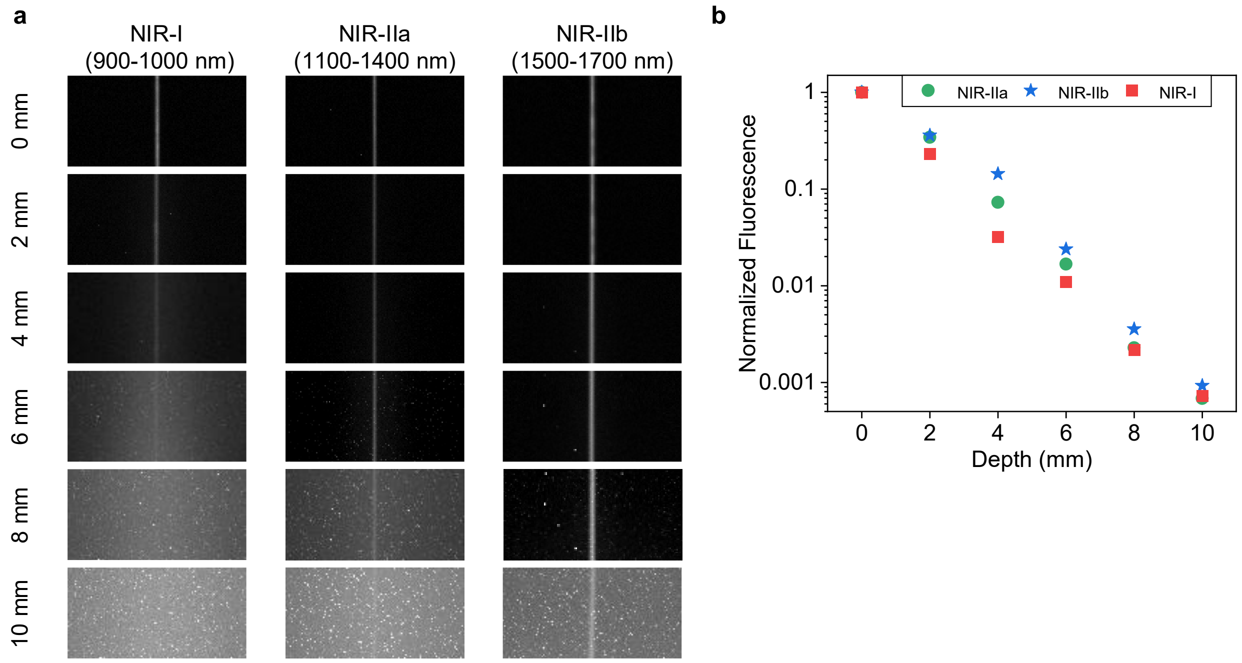

Figure S3. Phantom imaging in NIR-I, NIR-IIa and NIR-IIb sub-regions. (**a**) Imaging of a 100 μm diameter capillary filled with TSEH (emission collection: 900-1000nm, NIR-I), CTTIC (emission collection: 1100-1400 nm, NIR-IIa), or PbS (emission collection: 1500-1700 nm, NIR-IIb), which was immersed at different depths in 1% intralipid solution. To avoid differences caused by excitation, all probes were excited with an 808-nm laser. (**b**) Signal attenuation in the NIR-I, NIR-IIa, and NIR-IIb windows.

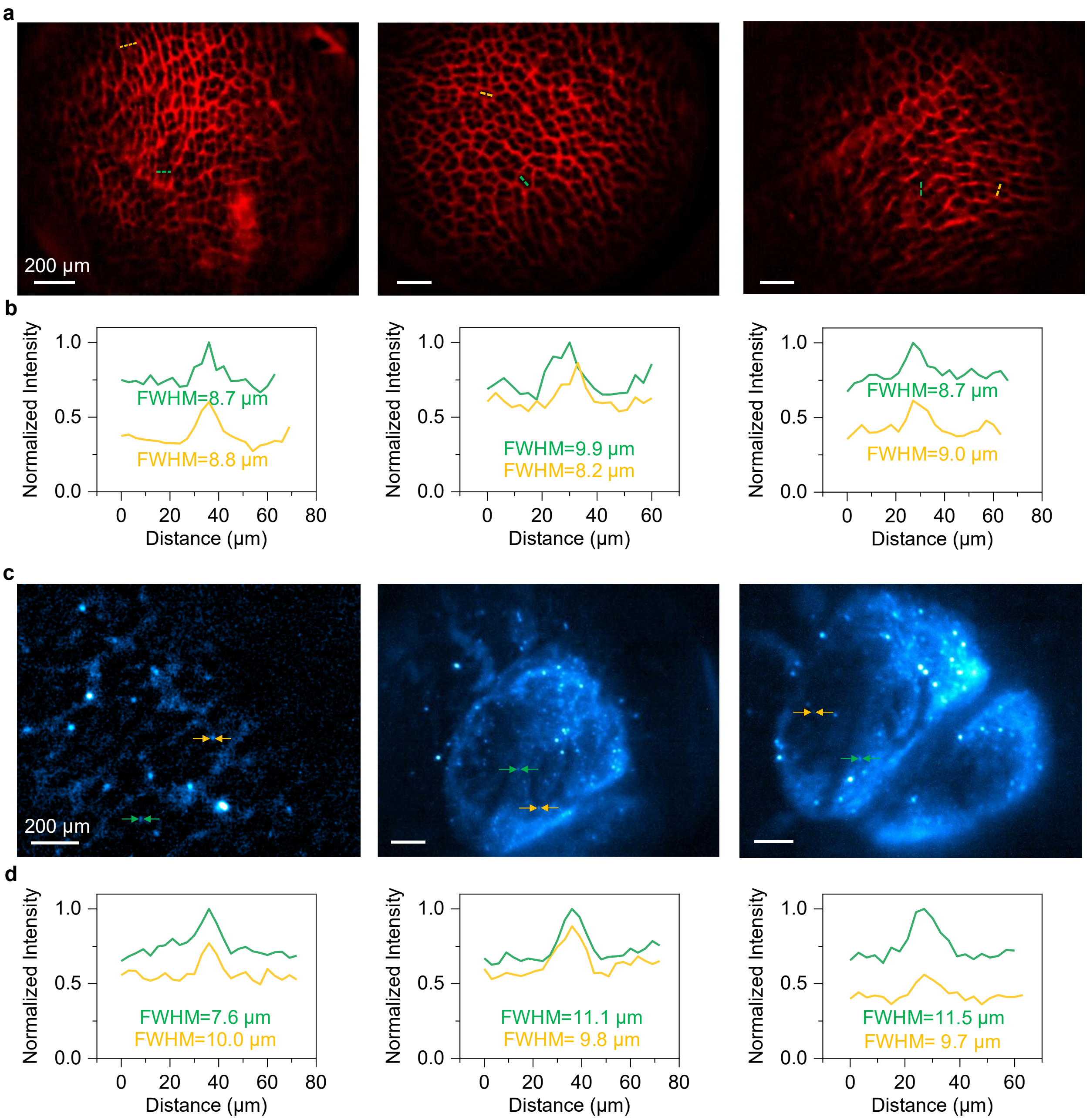

Figure S4. (**a**) A supplementary figure for Fig. 2c. (**b**) Normalized intensity profiles along the yellow or green lines in **(a)**. The lateral full width at half maximum (FWHM) values of the smallest intestinal vasculature using NIR-II microendoscopy were 8.68 ± 0.26 μm. (**c**) A supplementary figure for Fig. 4f-h. (**d**) Normalized Intensity profiles between the yellow or green arrows in **(c)**. The minimum lateral FWHM values of the CTTIC nanofluorophores imaged using NIR-II microendoscopy were 9.03 ± 1.01 μm, indicating that cellular resolution can be achieved. The average hydrodynamic size of CTTIC nanofluorophores was 60 nm.

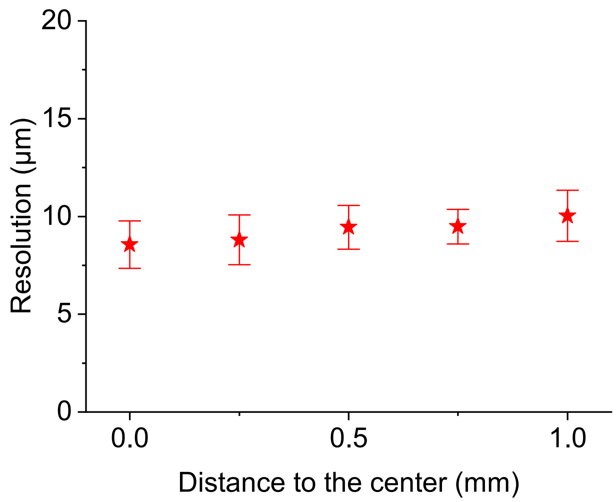

Figure S5. Variation of resolution within the field of view (FOV). The pentagrams represent the mean values of resolution, while error bars represent s.d. for n = 6.

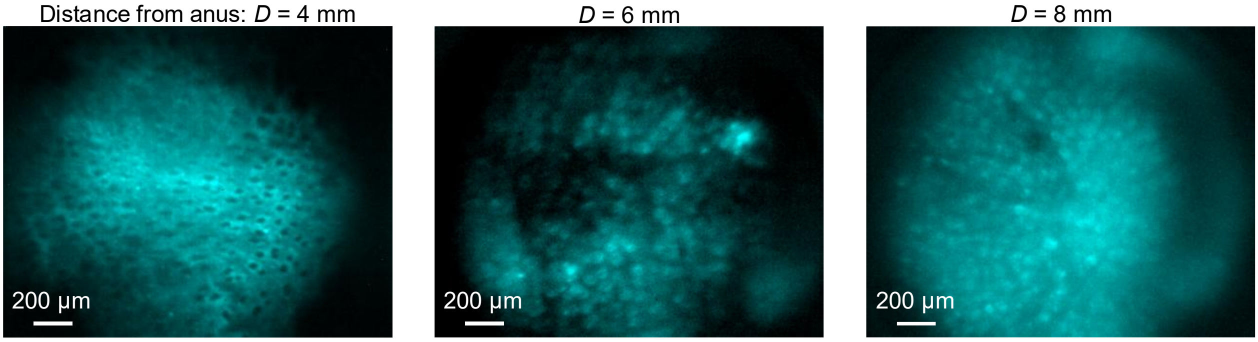

Figure S6. In vivo NIR-II microendoscopy of rectal crypts. Rectal crypts were observed in the region 4-8 mm from the anus, 15 minutes after injecting CNTIC-4F. A 975-nm laser was used for excitation, and fluorescence was filtered by a 1200-nm long-pass filter. The exposure time was 100 ms. Similar results for n > 3 independent experiments.

a

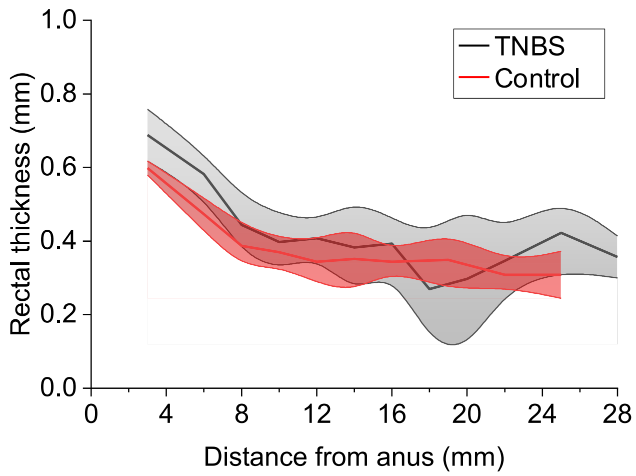

Figure S7. Rectal thickness of healthy mice and colitis-bearing mice. The rectal thickness of normal mice and 2,4,6-trinitrobenzene sulfonic acid (TNBS)-induced colitis-bearing mice were measured. The rectal thickness of healthy mice ranges from 300 μm to 600 μm, while that of colitis-bearing mice ranges from 200 μm to 800 μm. Solid lines represent the mean measurement values, and shaded areas indicate the standard deviation ranges. Three biological replicates were measured for each curve.

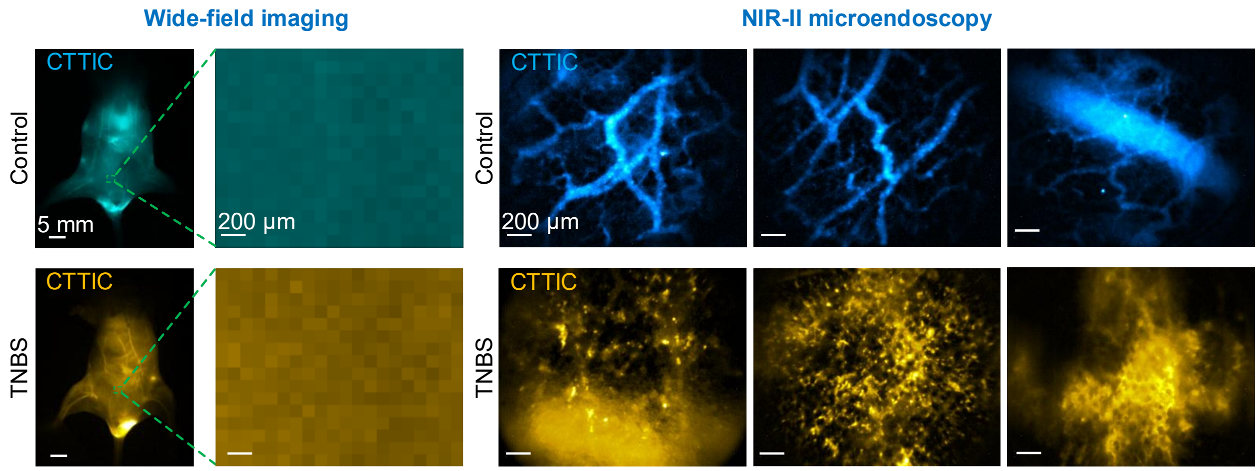

Figure S8. Comparison between NIR-II wide-field imaging and NIR-II microendoscopy at the same scale. The NIR-II wide-field imaging results in Fig. 5b were zoomed in to the same scale as the NIR-II microendoscopy results in Fig. 5c.

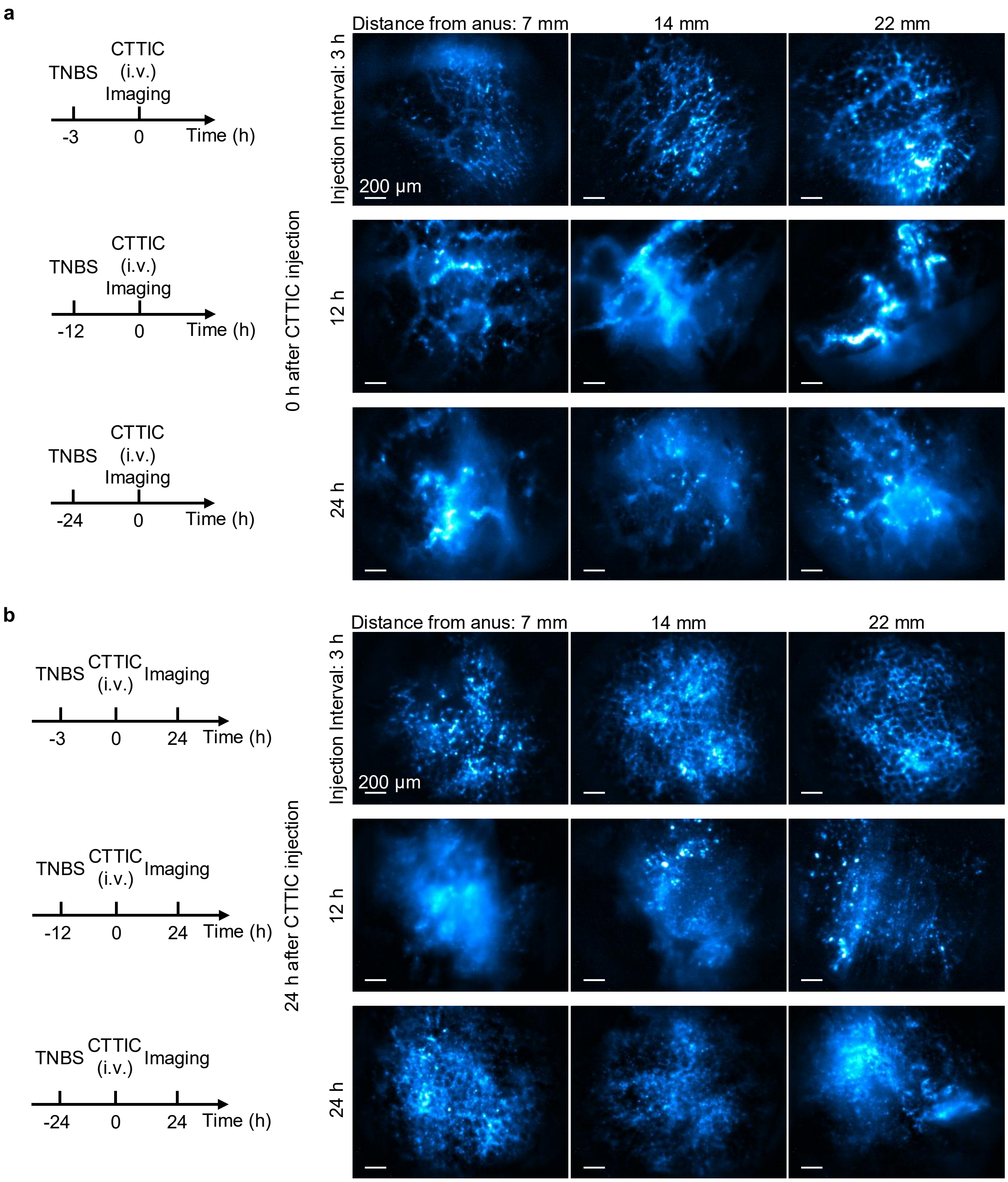

Figure S9. Longitudinal NIR-II microendoscopy of the rectal vasculature in mice with different injection intervals between TNBS and CTTIC. (**a**) In vivo NIR-II microendoscopy of CTTIC in the rectum at different distances from the anus was performed immediately after tail-vein injection of CTTIC (the injection intervals between TNBS and CTTIC were 3 h, 12 h, 24 h, respectively). The fluorescence signals showed CTTIC leakage in all the mice. (**b**) In vivo NIR-II microendoscopy of CTTIC in the rectum at different distances from the anus was performed 24 hours post tail vein injection of CTTIC (the injection intervals between TNBS and CTTIC were 3 h, 12 h, 24 h, respectively). The imaging results of normal mice in the control group can be found

in Fig. 5c,e. The fluorescence signals remained strong in all the mice. The exposure time was 100 ms.

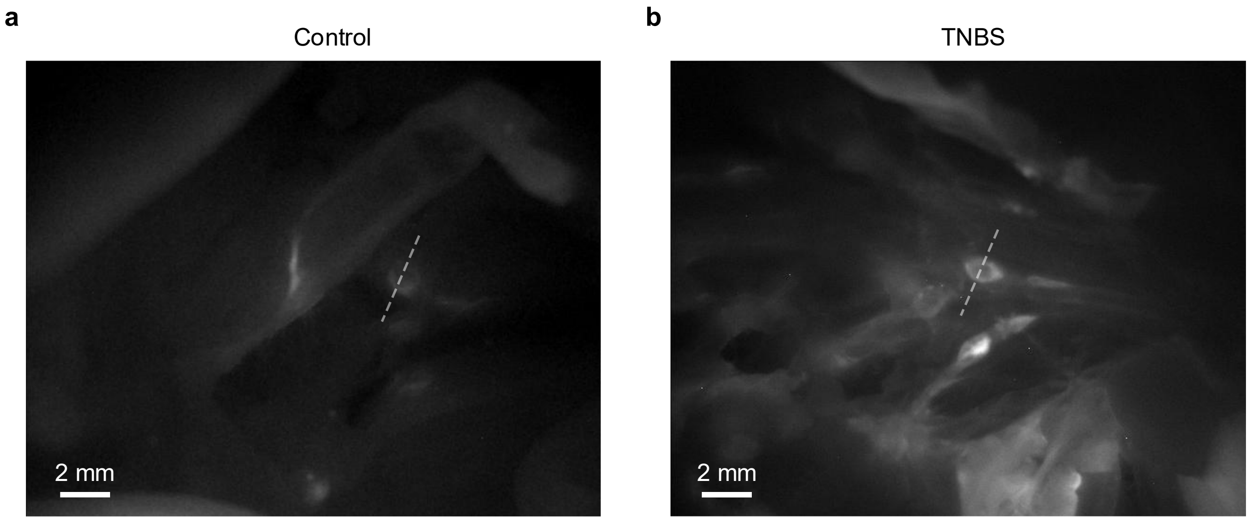

Figure S10. Ex vivo NIR-II imaging of lumbar LNs in healthy mice and colitis-bearing mice after dissection. Wide-field NIR-II imaging of the exposed lumbar LNs in (**a**) healthy mice and (**b**) TNBS-induced colitis-bearing mice as shown in Figs. 5 and 6. Healthy mice and colitis-bearing mice, injected with CTTIC intravenously, were sacrificed after all in vivo imaging and dissected to expose the lumbar LNs. Lumbar LN signals were measured after dissection to prevent the influence of the tissues above the lumbar LNs on the fluorescence signals. A 975-nm laser was used for CTTIC excitation, and its fluorescence was filtered by an 1100-nm long-pass filter and a 1400-nm short-pass filter. Scale bars, 2 mm.

Table S1. Comparison of different endoscopes.

Imaging type	Emission	Penetration depth	Working distance	FOV ($\mu m^2/\mu m$)	Horizontal resolution	Imaging through intestinal wall	Frame rate	Ref.
Confocal	502-712 nm	NA	650 μm	250 × 250	1 μm	No	30	1
Confocal	696-736 nm	NA	100 μm	300 × 300	0.87 μm	No	5	2
Confocal	NIR-I	NA	50 μm	350 × 350	3.2 μm	No	10	3
Confocal	502-633 nm or 673-800 nm	NA	NA	325	3.5 μm	No	10	4
Confocal	NIR-I	140 μm	NA	362 × 212	5 μm	No	5	5
Confocal	780-860 nm	300 μm	300 μm	330	1.55 μm	No	5	6
NIR-II endoscopy	>900 nm	NA	5 mm	4000	22 μm	No	NA	7
NIR-II microendoscopy	Up to 1700 nm	Submillimeter	0-5 mm	2100×1700	8.2 μm	Yes	10	**This work**

Table S2. Imaging conditions.

	Dyes	Excitation (Power)	Emission	Filters
Fig. 1d, Fig. 2c second row, Fig. 2d,e, Fig. 4e-h	CTTIC	915-nm laser (120 mW/cm^2)	1100 - 1400 nm	1100-nm long-pass filter
Fig. 2c first row	TSEH	785-nm laser (50 mW/cm^2)	900 - 1000 nm	900-nm long-pass filter, 1000-nm short-pass filter
Fig. 2c third row	PbS	915-nm laser (120 mW/cm^2)	1500 - 1700 nm	1500-nm long-pass filter
Fig. 3c	CNTIC-4F	975-nm laser (110 mW/cm^2)	1300 - 1600 nm	1300-nm long-pass filter
Fig. 3d-f, Fig. S6	CNTIC-4F	975-nm laser (110 mW/cm^2)	1200 - 1600 nm	1200-nm long-pass filter
Fig. 3f red channel	TSEH	785-nm laser (50 mW/cm^2)	1000 - 1200 nm	1000-nm long-pass filter, 1200-nm short-pass filter
Fig. 3f green channel	CTTIC	975-nm laser (110 mW/cm^2)	1200 - 1400 nm	1200-nm long-pass filter
Fig. 4d	CTTIC	975-nm laser (110 mW/cm^2)	1100 - 1400 nm	1100-nm long-pass filter
Fig. 5b,d, Fig. 6a, Fig. S10	CTTIC	975-nm laser (110 mW/cm^2)	1100 - 1400 nm	1100-nm long-pass filter, 1400-nm short-pass filter
Fig. 5c,e,g, Fig. S9, Fig. 6c	CTTIC	915-nm laser (120 mW/cm^2)	1100 - 1400 nm	1100-nm long-pass filter, 1400-nm short-pass filter
Fig. 6a,c, Fig. S1b	PbS	808-nm laser (100 mW/cm^2)	1500 - 1700 nm	1500-nm long-pass filter

References:

1. Kim, P. et al. In vivo wide-area cellular imaging by side-view endomicroscopy. *Nat Methods* **7**, 303-305 (2010).
2. Duan, X. et al. Visualizing epithelial expression of EGFR in vivo with distal scanning side-viewing confocal endomicroscope. *Sci Rep* **6**, 37315 (2016).
3. Hwang, K. et al. Handheld endomicroscope using a fiber-optic harmonograph enables real-time and in vivo confocal imaging of living cell morphology and capillary perfusion. *Microsyst Nanoeng* **6**, 72 (2020).
4. Yoo, S.W. et al. Development of Dual-Scale Fluorescence Endoscopy for In Vivo Bacteria Imaging in an Orthotopic Mouse Colon Tumor Model. *Appl. Sci.* **10**, 844 (2020).
5. Piyawattanametha, W. et al. In vivo near-infrared dual-axis confocal microendoscopy in the human lower gastrointestinal tract. *J Biomed Opt* **17**, 021102 (2012).
6. Wang, J. et al. Near-infrared probe-based confocal microendoscope for deep-tissue imaging. *Biomed Opt Express* **9**, 5011-5025 (2018).
7. Suo, Y. et al. NIR-II Fluorescence Endoscopy for Targeted Imaging of Colorectal Cancer. *Adv Healthc Mater* **8**, e1900974 (2019).
